# Supplementary material for: Apple Pomace and Performance, Intestinal Morphology and Microbiota of Weaned Piglets—A Weaning Strategy for Gut Health?
Source: Microorganisms. 2021 Mar 10;9(3):572. doi: 10.3390/microorganisms9030572 (PMC7998770; doi:10.3390/microorganisms9030572)
Supplement: Supplementary file 1 [file microorganisms-09-00572-s001.zip › microorganisms-1119071-supplementary.docx]

**Supplementary file: Apple pomace and performance, intestinal morphology and microbiota of weaned piglets – a weaning strategy for gut health?**


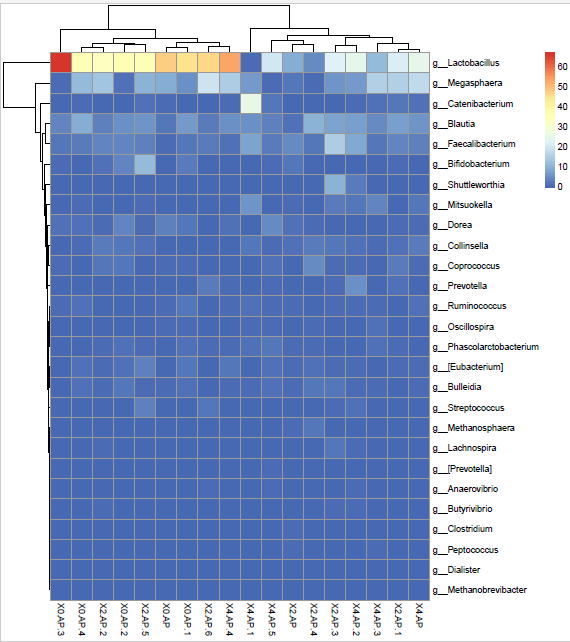


Figure S1: Genus abundance Heat Map for faeces on the 8 post-weaning day. Genera with at least 0.5% of relative abundance in one sample for each diet were used. X0.AP: 0%AP diet; X2.AP: 2%AP diet; X4.AP: 4%AP diet.


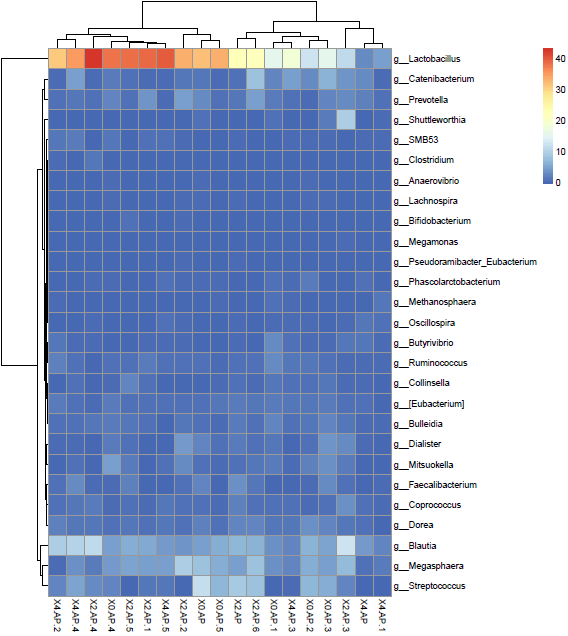


Figure S2: Genus abundance Heat Map for faeces on the 28 post-weaning day. Genera with at least 0.5% of relative abundance in one sample for each diet were used. X0.AP: 0%AP diet; X2.AP: 2%AP diet; X4.AP: 4%AP diet.
